# Lovastatin Modulates Glycogen Synthase Kinase-3β Pathway and Inhibits Mossy Fiber Sprouting after Pilocarpine-Induced Status Epilepticus

**DOI:** 10.1371/journal.pone.0038789

**Published:** 2012-06-26

**Authors:** Chun-Yao Lee, Thomas Jaw, Huan-Chin Tseng, I-Chun Chen, Horng-Huei Liou

**Affiliations:** 1 Department of Pharmacology, College of Medicine, National Taiwan University, Taipei, Taiwan; 2 Department of Neurology, National Taiwan University Hospital, Taipei, Taiwan; Massachusetts General Hospital/Harvard Medical School, United States of America

## Abstract

This study was undertaken to assay the effect of lovastatin on the glycogen synthase kinase-3 beta (GSK-3β) and collapsin responsive mediator protein-2 (CRMP-2) signaling pathway and mossy fiber sprouting (MFS) in epileptic rats. MFS in the dentate gyrus (DG) is an important feature of temporal lobe epilepsy (TLE) and is highly related to the severity and the frequency of spontaneous recurrent seizures. However, the molecular mechanism of MFS is mostly unknown. GSK-3β and CRMP-2 are the genes responsible for axonal growth and neuronal polarity in the hippocampus, therefore this pathway is a potential target to investigate MFS. Pilocarpine-induced status epilepticus animal model was taken as our researching material. Western blot, histological and electrophysiological techniques were used as the studying tools. The results showed that the expression level of GSK-3β and CRMP-2 were elevated after seizure induction, and the administration of lovastatin reversed this effect and significantly reduced the extent of MFS in both DG and CA3 region in the hippocampus. The alteration of expression level of GSK-3β and CRMP-2 after seizure induction proposes that GSK-3β and CRMP-2 are crucial for MFS and epiletogenesis. The fact that lovastatin reversed the expression level of GSK-3β and CRMP-2 indicated that GSK-3β and CRMP-2 are possible to be a novel mechanism of lovatstain to suppress MFS and revealed a new therapeutic target and researching direction for studying the mechanism of MFS and epileptogenesis.

## Introduction

Temporal lobe epilepsy (TLE) is the most prevalent symptom in patients who are diagnosed with epilepsy. TLE is mainly caused by abnormal neuronal circuitry changes in the hippocampal formation, which is vulnerable to excitotoxicity and easily generates a focus of spontaneous recurrent seizures (SRS). Several pathological features have been found in the hippocampal formation under epileptic condition, including hippocampal sclerosis, massive amount of neuronal loss caused by either necrosis or apoptosis, neurogenesis, neuro-inflammation, granule cell dispersion (GCD) and mossy fiber sprouting (MFS) [1]. Among all these features caused by epileptic injury, MFS in the dentate gyrus (DG) is the most important index that is highly correlated with the frequency of SRS and the severity of TLE [2], [3].

Mossy fibers are the axons of granule cells in the DG targeting to pyramidal cells in the CA3 region. During seizure spreading, over-excitability induces large amount of glutamate release from the nerve terminals of mossy fibers and evoked abnormal discharges on the CA3 pyramidal cells, which further exaggerates the seizure activities and neuronal damages [4]. After the initial epileptic injury, mossy fibers lose their targets by the massive neuronal death in the CA3 area, form incorrect synaptic connections on the dendrites of granule cells themselves in the inner molecular layer of the DG, causing a recurrent circuit with hyper-excitabilities [5], [6]. Also, the reduction of MFS is beneficial to slower the process of epileptogenesis [6]. The mechanism of MFS is known to related to NMDA receptor [7], new protein synthesis [8], c-fos signaling [9], neuro-inflammatory factors [10] and mammalian target of rapamycin (mTOR) pathway [11] by several pharmacology-based studies. However, the underlying mechanism of MFS is still not well-understood.

Statins have been taken for non-traditional uses (lipid-lowering agents) in the treatment of neurological diseases, including stroke and brain trauma. Clinical trials find that statins reduce the risk of stroke [12]–[14] via Akt and its downstream signaling targets [15], [16]. Statins are also reported to restrain kainic acid-induced seizures and the associated neuro-inflammation and hippocampal cell death [10], [17].

Besides reducing neuro-inflammation, statins also exert neuroprotective effect by regulating glycogen synthase kinase-3β (GSK-3β) pathway [15], [16]. GSK-3β is one of the downstream genes of Akt, which is a key molecule in neuronal polarity determination. Inactivation or down-regulation of GSK-3β enhances axonal elongation and branching. Active form of GSK-3β reduces axonal growth by phosphorylation of collapsin response mediator protein-2 (CRMP-2) [18], which is known to contributes to axonal pathfinding [19]. Over-expression of CRMP-2 in hippocampal neurons induces the formation of multiple axons and elongation of the primary axons [20].

In the present study, we are interested in investigating whether lovastatin affects the expression of GSK-3β and CRMP-2 in the TLE animal model and inhibits MFS. Our results showed that the expression level of GSK-3β and CRMP-2 was increased after in TLE animal model and the administration of lovastatin reversed this alteration and effectively reduced the extent of MFS in both DG and CA3 regions. These findings unveiled a novel mechanism of lovastatin on MFS.

## Materials and Methods

### Animal Model of TLE

The pilocarpine-induced status epilepticus (SE) animal model of chronic epilepsy in rats replicates several characteristics of human TLE [2], [21]. Experimental animals were housed in polycarbonate boxes (four rats per box) in accordance with the *Guide for the Care and Use of Laboratory Animals* by the US National Institutes of Health. They were maintained in a controlled atmosphere with a 12 h dark/light cycle (lights off at 7∶00 PM), a temperature of 22±2°C and 50–70% humidity with free access to pelleted feed and fresh tap water. All efforts were made to minimize animal suffering and to reduce the number of animal used. The experiment was approved by the Animal Ethics Committee of National Taiwan University. Male Wistar rats (around 4–6 weeks old) were injected with pilocarpine (300–380 mg/kg, INTRAPERITONEAL, i.p.) to induce SE. The behavioral seizure was evaluated according to Racine’s score [22]. SE was defined as continuous convulsions with a score of 4 to 5 for at least 1 hour and terminated by pentobarbital (25–30 mg/kg, i.p). The details of TLE animal model were previously described [23].

### Western Blotting

Rat brain tissues were collected from three groups: (1) control group, (2) 3 and 7 days after SE induction and (3) 3 and 7 days after SE induction with lovastatin injection. Protein samples were harvested by lysing hippocampi into lysis buffer supplemented with protease inhibitor mixture. Protein concentrations were determined using a Bradford protein assay kit (Bio-Rad, Hercules, CA, USA). Typically, an aliquot of 10 µg of protein was loaded into each well of 12% SDS-polyacrylamide gel and separated by electrophoresis. The proteins were then transferred to nitrocellulose membrane then incubated with indicated antibodies using standard protocols. Antibodies against GSK-3β, phospho-GSK-3β (Ser 9) were products from Epitomics (Burlingame, CA, USA). Antibodies against CRMP-2 were purchased from Cell Signaling Technology (Danvers, MA, USA). An ECL Plus western blotting detection system (Amersham Biosciences, Little Chalfont, UK) was used to obtain western blotting signals, and the autoradiographs were scanned. The intensity of immunoblot signals was analyzed by Image J software (http://rsbweb.nih.gov/ij/).

### Histology

The animals were kept for 1 month after SE induction and sacrificed for histological observation during the progression of MFS. After deep anesthesia with pentobarbital (80 mg/kg, i.p.), animals were perfused transcardially with 200 ml of sodium sulfide perfusion medium (2.925 g Na_2_S, 2.975 g NaH_2_PO4 in 500 ml H_2_O) followed by 200 ml 4% paraformaldehyde. The rat brains were dissected from the cranium and fixed in a glass vial filled with 4% paraformaldehyde solution for overnight post-fixation. After fixation, the brains were transferred to phosphate buffer solution.

The details of Timm’s stain are referred from our previous publication [23]. The brains were transferred in a 30% sucrose solution for dehydration until the brains sank to the bottom of the vials. For histology, 30-µm-thick brain tissue contained hippocampus was sectioned by a freezing microtome and then attached on coated glass slides. We stained one in every six sections, amounting to approximately 36 sections for each rat, and calculated the Timm’s scores of these stained sections based on the following procedures. The sections were developed in the dark for 45 minutes in a solution of 50% Arabic gum (120 ml), 10 ml citric acid (51 g/100 ml H_2_O), 10 ml sodium citrate (47 g/100 ml H_2_O), 3.47 g hydroquinone in 60 ml, and 212.25 mg AgNO_3_. After washing, the slices were dehydrated in graded alcohol, cleared in xylene, and mounted on slides with Permount. The Timm’s score in DG was evaluated by the following criteria: score 0, no granules noted between crest and tips in the supragranular region; score 1, occasional granules in the supragranular region occurring in patchy distribution; score 2, numerous granules in the supragranular region occurring in patchy distribution; score 3, granules in the supragranular region occurring in near-continuous distribution; score 4, highly concentrated band of granules appearing either in continuous or near continuous distribution; score 5, continuous dense laminar band of granules from the crest to the tip of the dentate. In certain experiments, we also observed the Timm’s score in CA3 area. Similarly, there are five scores in CA3: score 0, No granules in the stratum pyramidale or stratum oriens along any portion of the CA3 subregion; score 1, occasional granules in the stratum pyramidale or stratum oriens occurring in discrete bundles; score 2, occasional to moderate granules in the stratum pyramidale or stratum oriens; score 3, prominent granules in the stratum pyramidale or stratum oriens; score 4, prominent granules in the stratum pyramidale or stratum oriens occurring in near-continuous distribution along the entire CA3 region; score 5, continuous or near-continuous dense laminar band of granules in the stratum pyramidale or stratum oriens along the entire CA3 region [24]. Timm’s score was calculated by each section, averaged from all the scores of stained sections and taken as one rat’s score for DG and CA3 accordingly. The stained sections were analyzed for each rat by one of the authors (CYL) blind to experimental groups and analyzed by using a semi-quantitative scale for supragranular layer of the DG and terminal sprouting CA3 pyramidal cell region.

GCD was evaluated by Nissl stain. The slices were obtained as abovementioned methods. Slices were immersed in 0.1% cresyl violate solution, and the distances from the inner (hilar) border of the granule cell layer to the outer border of the most distal granule cell somata of the DG (thickness of GCD) were measured and analyzed referring to previous report [25].

### Electrophysiology

The electrophysiology was performed based on our previous study [26]. To performing whole-cell patch-clamp recording, male Wistar rats (4–6 weeks old) were decapitated under deep anesthesia with halothane inhalation and the brains quickly moved to ice-cold oxygenated cutting solution containing (in mM): 125 NaCl, 2.5 KCl, 0.5 CaCl_2_, 5 MgCl_2_, 26 NaHCO_3_, 15 glucose and aerated to pH 7.4 with 95% O_2_-5% CO_2_. Coronal brain slices (300 µm) containing DG were cut by microslicer (DTK-1000, Dosaka, Kyoto, Japan) in cutting solution and then transferred to a holding chamber with artificial cerebrospinal fluid (ACSF) containing (in mM): 125 NaCl, 2.5 KCl, 2 CaCl_2_, 1 MgCl_2_, 26 NaHCO_3_, 15 glucose and aerated with 95% O_2_ - 5% CO_2_. Brain slices were then maintained at room temperature (23±2°C) for at least 1 h before recording. The brain slice was then transferred to the recording chamber, held submerged and superfused continuously with ACSF at a flow rate of 1–2 ml/min for recording at room temperature (23±2°C). Evoked NMDA receptor-mediated EPSCs (eEPSC_NMDA_) were recorded in Mg^2+^-free ACSF in the presence of bicuculline methioide (50 µM), 6-cyano-7-nitroquinoxaline-2,3-dione (CNQX, 10 µM), and D-serine (10 µM) at a holding potential of −70 mV. Patch electrodes were pulled from standard-walled borosilicate glass capillaries (CSF-150, Warner Instrument, USA) by micropipette puller (P97, Sutter Instrument, USA) with a resistance of 3–8 MΩ, then filled with a CsCl-based internal solution containing (in mM): 140 CsCl, 9 NaCl, 1 MgCl_2_, 1 EDTA, 10 HEPES, 5 QX-314, 2 Mg-ATP, 0.3 Na-GTP (pH was adjusted to 7.3 with 1 N CsOH). The granule cells in DG were visually identified by using an upright infra-red microscope fitted with a water-immersion lens (Olympus, Tokyo, Japan). Whole-cell patch-clamp recording was made from the granule cells in DG of rat brain slices with an Axopatch 200B amplifier (Axon Instruments, Foster City, CA, USA). eEPSC_NMDA_ were evoked by a stimulation glass pipette filled with 3 M NaCl solution was placed on the molecular layer of DG with a stimulator (S-48, Grass-Telefactor, USA) and isolation unit (A.M.P.I., Jerusalem, Israel) with a frequency of 0.1 Hz. eEPSC_NMDA_ were quantified by measuring peak amplitudes of average responses. The data was acquired by Clampex 9.0 software (Axon Instruments, Foster City, CA, USA), stored in the hard drive and analyzed by Clampfit 9.0 (Axon Instruments, Foster City, CA, USA).

### Drugs

Bicuculline methioide, dexamethasone, L-NAME, lovastatin, MK-801, and pentobarbital were purchased from Sigma (St. Louis, MO, USA). CNQX and D-serine were purchased from Tocris (Bristol, UK). Drugs for injection were dissolved in 0.9% NaCl solution and directly administrated subcutaneously 3 hours after intraperitoneal injection of pentobarbital. Drugs for electrophysiology were dissolved in double distilled water except CNQX were dissolved in 100% DMSO as stock solution (the final concentration of DMSO in ACSF was less than 0.1%). The dosages for investigation were summarized in Table 1.

**Table 1:** Drug administration regimen.

Drugs	Dosage (s.c.)
Lovastatin (HMG-CoA inhibitor)	20 mg/kg
MK-801 (NMDA receptor blocker)	0.5 mg/kg
L-NAME (iNOS inhibitor)	20 mg/kg
Dexamethasone (Steroid)	20 mg/kg

All the drugs were injected subcutaneously after SE induction.

### Statistics

Statistical differences were established by Student’s *t*-test, one-way ANOVA and post-hoc Mann-Whitney U test. Data were expressed as a mean ± SE, and *P*<0.05 was taken to indicate statistical significance. The n values referred the number of animal used.

## Results

**Figure 1:**
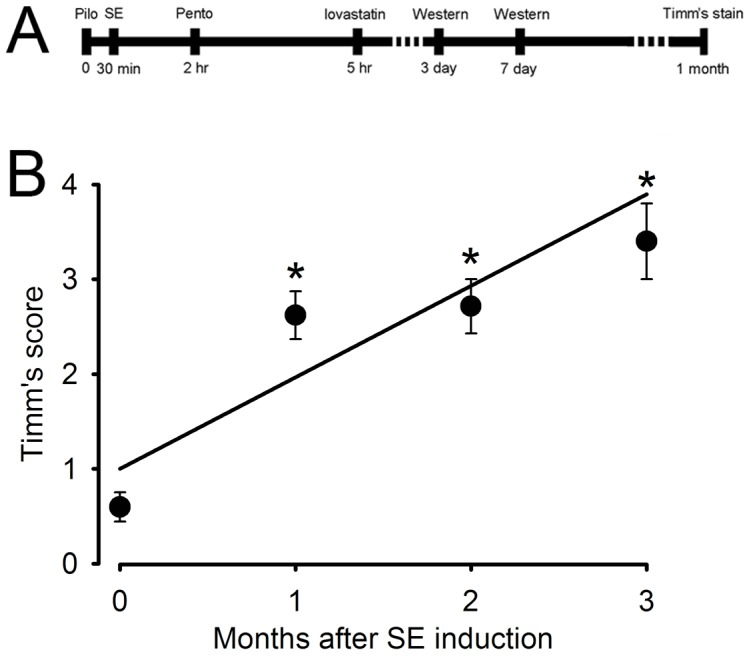
The experimental design and Timm’s score recorded from different time points. (A) The experimental design of TLE animal model and drug administration. (B) Timm’s score recorded from control and 1 to 3 months after SE induction. 1 month after SE induction, the Timm’s score was significantly increased compared with control group and show an increasing tendency with time. (*compared with control group).

It is known that GSK-3β regulates axonal growth and neuronal polarity through phosphorylating CRMP-2 [18], therefore we examined the expression level and phosphorylation state of GSK-3β and CRMP-2 from control, SE and SE+lovastatin groups, respectively, by western blotting method. We administrated lovastatin (20 mg/kg) to the rats 3 hours after terminating SE by pentobarbital. The experimental regimen is illustrated in Figure 1A. To determine a proper observation time point of MFS, we evaluated the Timm’s score from 1 to 3 months after SE induction. The Timm’s score after SE induction was 0.60±0.15 in control group (n = 16), 2.62±0.25 in 1 month SE group (n = 16, *P*<0.001), 2.71±0.29 in 2 months SE group (n = 7, *P*<0.001), and 3.40±0.40 in 3 months SE group (n = 5, *P*<0.001)(one-way ANOVA and post-hoc Mann-Whitney U test, regression constant, 0.71) (Figure 1B). Therefore, to minimize the animal suffering and consumption and to reduce the experimental duration, we chose 1 month as our observation time point to record Timm’s score.

### The Expression and Phosphorylation Pattern of GSK-3β after Seizure Induction and the Administration of Lovastatin

**Figure 2:**
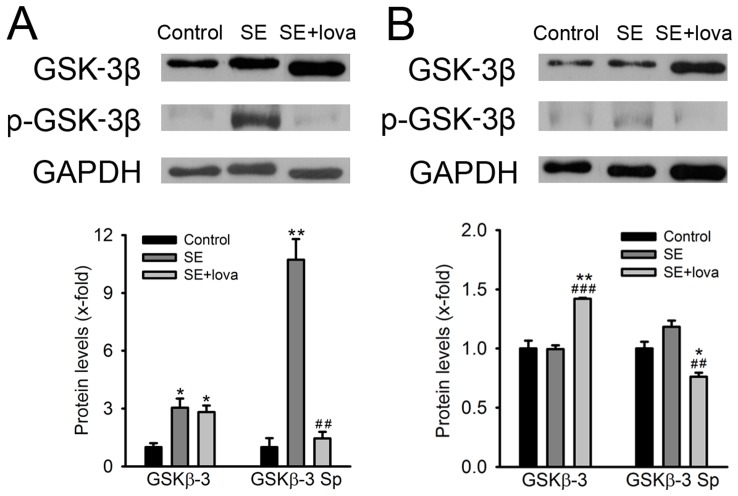
Lovastatin significantly alter the expression and phosphorylation pattern of GSK-3β at day 3 and 7 after pilocarpine-induced SE. The GSK-3β protein expression and phosphorylation levels from control, SE and SE+statin rat hippocampus was determined by western blot. pGSK-3β indicate the serine phosphorylation of GSK-3β. GAPDH was used as loading control. Lower panel show quantification of the relative protein amount of GSK-3β and serine phosphorylation level of GSK-3β. (A) The expression level of GSK-3β at day 3 after SE. (B) The expression level of GSK-3β at day 7 after SE. (*compared with control group; #compared with SE group).

We firstly examined the total expression level of GSK-3β. At day 3 after SE induction, the amount of GSK-3β in both SE and SE+lovastatin group were about three times higher than control group (control, 1.00±0.20; SE, 3.04±0.48; SE+lovastatin 2.82±0.35, *P*<0.05, n = 3). At day 7 after SE, the amount of GSK-3β in SE is similar as control but significantly higher in SE+lovastatin group (control, 1.00±0.07; SE, 0.99±0.03; SE+lovastatin, 1.42±0.01, *P*<0.05, n = 3). Furthermore, we examined the states of serine phosphorylation of GSK-3β (pGSK-3β). At day 3 after SE, the expression of pGSK-3β in SE group was about ten times increased than control and was significantly reversed in SE+lovastatin group (control, 1.00±0.47; SE, 10.73±1.07; SE+lovastatin, 1.45±0.34, *P*<0.05, n = 3). At day 7 after SE, the expression of pGSK-3β slightly increased in SE group and was significantly decreased in SE+lovastatin group (control, 1.00±0.06; SE, 1.18±0.05; SE+lovastatin, 0.76±0.03, *P*<0.05, n = 3) (Student’s *t*-test)(Figure 2).

### The Expression Level of CRMP-2 after Seizure Induction and the Administration of Lovastatin

**Figure 3:**
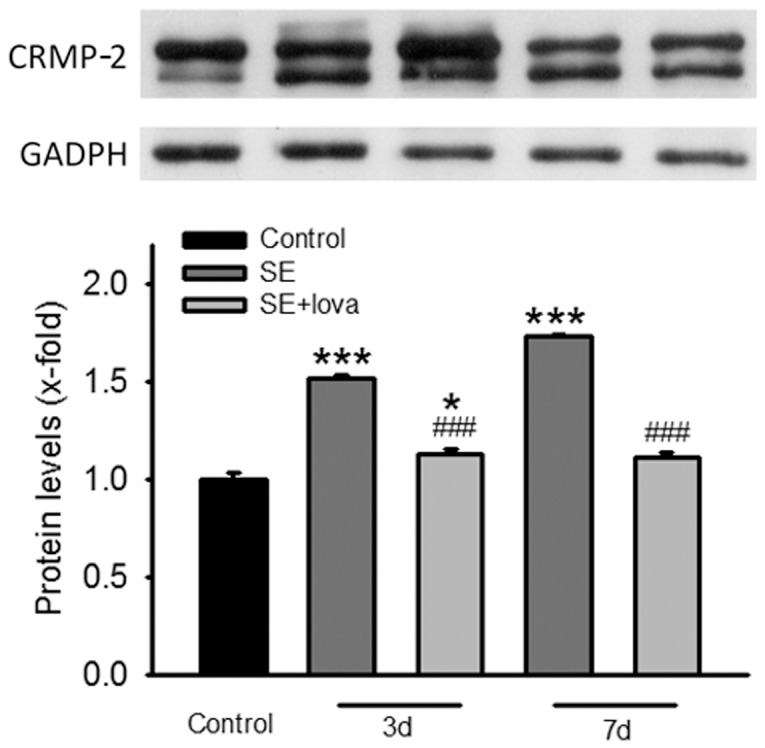
Lovastatin significantly alter the expression pattern of CRMP-2 at day 3 and day 7 after pilocarpine-induced SE. The CRMP-2 protein expression levels from control, SE and SE+statin was determined by western blot. GAPDH was used as loading control. Lower panel show quantification of the relative protein amount of CRMP-2. (*compared with control group; #compared with SE group).

In the next, we examined the expression level CRMP-2, which is the downstream signal of GSK-3β. At day 3 after SE, the expression level of CRMP-2 of SE group is higher than control (1.52±0.03, *P*<0.05, n = 3) but the level of CRMP-2 in SE+lovastatin group was not significantly increased (1.12±0.05, *P*>0.05, n = 3). At day 7 after SE, the amount of CRMP-2 in SE group is higher than control (1.71±0.02, *P*<0.05, n = 3) but the amount of CRMP-2 in SE+lovastatin group was not significant changed (1.11±0.05, *P*>0.05, n = 3) (Student’s *t*-test) (Figure 3).

### Lovastatin Inhibited MFS

**Figure 4:**
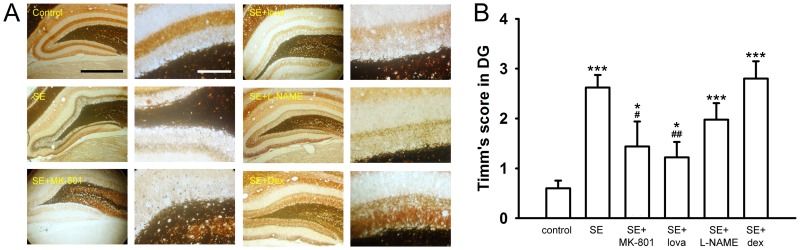
Timm’s stain under different treatments in the DG. (A) The Timm’s score was significantly increased after SE induction. MK-801 and lovastatin significantly decreased Timm’s score after SE induction. However, L-NAME and dexamethasone did not show any effect. Scale bar, 200 µm in the left panels and 100 µm for the right panels. (B) Summary data showing the mean Timm’s score between control and experimental groups in the DG. (*compared with control group; #compared with SE group).

**Figure 5:**
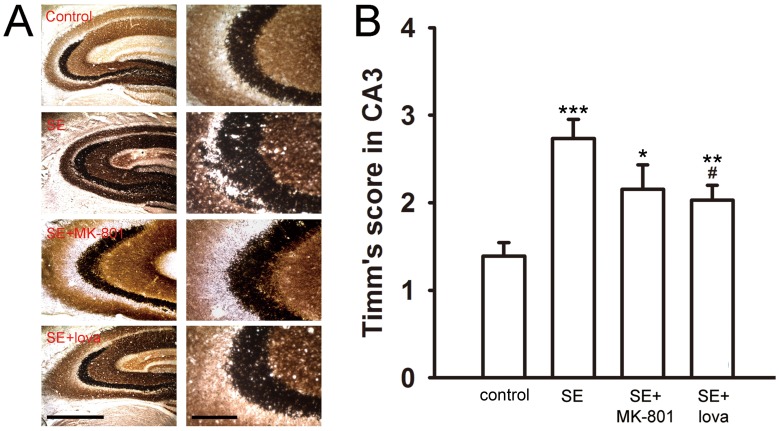
Timm’s stain under different treatments in the CA3. (A) The Timm’s score was significantly increased after SE induction. MK-801 and lovastatin significantly decreased Timm’s score after SE induction. Scale bar, 200 µm in the left panels and 100 µm for the right panels. (B) Summary data showing the mean Timm’s score between control and experimental groups in the CA3. (*compared with control group; #compared with SE group).

After drug administrated, we recorded the Timm’s score 1 month after SE induction to evaluate the severity of MFS. In our result, MK-801 and lovastatin significantly reduced Timm’s score but L-NAME and dexamethasone did not show the similar effect. The Timm’s score was 0.60±0.15 in control group (n = 16), 2.62±0.25 in SE group (n = 16, *P*<0.001, compared with control group), 1.44±0.56 in SE+MK-801 group (n = 6, *P*<0.05, compared with control group; *P*<0.05, compared with SE group), 1.22±0.31 in SE+lovastatin group (n = 12, *P*<0.05, compared with control group; *P*<0.01, compared with SE group), 1.98±0.33 in SE+L-NAME group (n = 10, *P*<0.01, compared with control group; *P*>0.05, compared with SE group), and 2.80±0.35 in SE+dexamethasone group (n = 5, *P*<0.001, compared with control group; *P*>0.05, compared with SE group)(Figure 4, one-way ANOVA and post-hoc Mann-Whitney U test). We next observed whether we could find the similar phenomenon in CA3 area. The Timm’s score was 1.39±0.16 in control group (n = 17), 2.74±0.22 in SE group (n = 15, *P*<0.001, compared with control group), 2.15±0.28 in SE+MK-801 group (n = 6, *P*<0.05, compared with control group; *P*>0.05, compared with SE group), 2.03±0.17 in SE+lovastatin group (n = 12, *P*<0.01, compared with control group; *P*<0.05, compared with SE group), respectively (Figure 5, one-way ANOVA and post-hoc Mann-Whitney U test). This result showed that lovastatin inhibited axonal sprouting in both DG and CA3 areas after SE induction.

### Lovastatin did not Behave as an NMDA Receptor Blocker

**Figure 6:**
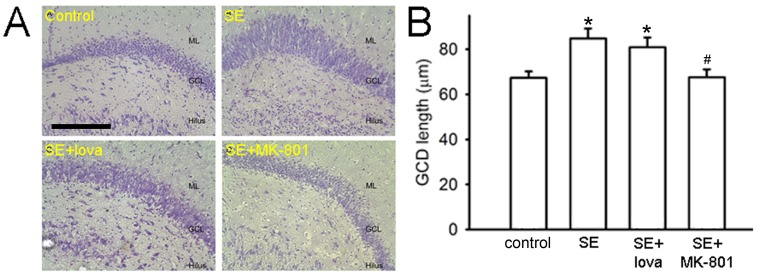
Nissl stain under different treatments in the DG. (A) After SE, the thickness of granule cell layer was significant increased. After SE induction, MK-801 significantly decreased the thickness of granule cell layer but lovastatin did not. Scale bar, 100 µm. (B) Summary data showing the mean thickness of granule cell layer between control and experimental groups in the DG. (*compared with control group; #compared with SE group).

At last, we tested whether lovastatin behaves as a NMDA receptor blocker (like MK-801) to reduce Timm’s score. It is known that 1 month after SE induction, the thickness of granule cell layer in the DG is increased (GCD) and could be prevented by MK-801 [27]. If lovastatin itself is an NMDA receptor blocker, GCD should be accordingly decreased. In our result, the thickness of granule cell layer in the DG is 67.3±2.8 µm in control group (n = 6), 84.9±4.3 µm in SE group (n = 18, *P*<0.05 compared with control group), 80.9±4.3 µm in SE+lovastatin group (n = 12, *P*<0.05 compared with control group; *P*>0.05 compared with SE group), 67.6±3.5 µm in SE+ MK-801 group (n = 6, *P*>0.05 compared with control group; *P*<0.05 compared with SE group) (Figure 6, Student’s *t*-test). The result proposed that lovatstatin did not show any effect on GDC as MK-801 did.

**Figure 7:**
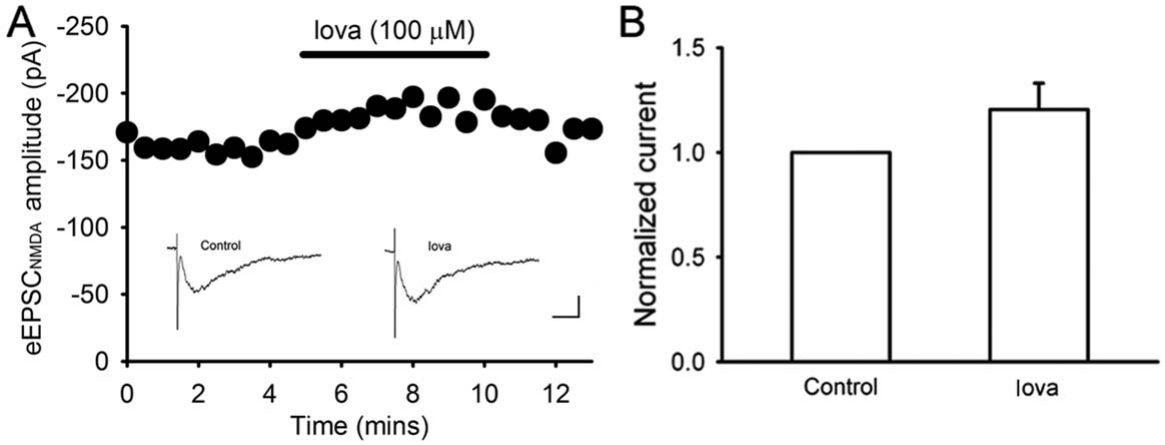
The effect of lovastatin on the eEPSC_NMDA_. (A) The eEPSC_NMDA_ was not significantly altered by lovatstatin (100 µM) application. Scale bar, 20 ms, 100 pA. (B) Summary data showing the mean amplitude of the eEPSC_NMDA_ between control and experimental groups in the DG.

Furthermore, we directly recorded eEPSC_NMDA_ from the granule cells in the DG to test whether lovastatin acts on NMDA receptor. Lovastatin (100 µM) was bath applied to the hippocampal brain slices and the amplitude of eEPSC_NMDA_ were recorded. According to our observation, lovastatin did not significantly reduce the mean amplitude of eEPSC_NMDA_. The mean amplitude of eEPSC_NMDA_ after application of lovastatin was 120.4±12.6 (% of control, n = 3, *P>*0.05, Student’s *t*-test)(Figure 7), indicating that lovastatin did not directly affect NMDA receptor-mediated synaptic transmission in the DG. The present results demonstrated that lovastatin did not affect NMDA receptors.

## Discussion

In the present study, we found an increased expression of GSK-3β, pGSK-3β and CRMP-2 after SE induction. Application lovastatin reversed the alteration of pGSK-3β/GSK-3β and CRMP-2 and inhibited MFS. Therefore we propose that lovastatin inhibited MFS by regulating the expression and phosphorylation state of GSK-3β and CRMP-2 other than anti-inflammatory and NMDA receptor blocking effect.

### Lovastatin Reversed the Increase of pGSK-3β/GSK-3β Ratio and CRMP-2 after SE Induction

Statins are reported to activate the PI3K/Akt pathway, affect neurogenesis, delay neuronal death, improve spatial learning and associated with therapeutic improvement after traumatic brain injury in the DG [15], [16]. GSK-3β is one of the genes that responsible to neuronal survival and axonal growth. The activity of GSK-3β is negatively regulated by N-terminal phosphorylation of Ser 9 [28]. The phosphorylation state of GSK-3β controls neuronal survival and axonal growth [29] through regulating CRMP-2 [18]. Our data showed that the expression of GSK-3β was increased and highly phosphorylated at 3 days after SE induction (Figure 2A), accompanied with an increase of CRMP-2 expression (Figure 3). This could lead to axonal growth and guidance in the formation of MFS. When applying lovastatin, the pGSK-3β was decreased but the total GSK-3β was not change, indicating that lovastatin mostly reduced pGSK-3β and decreased pGSK-3β/GSK-3β ratio, further to inactivate CRMP-2 and to inhibit MFS. Although SE-induced phosphorylation of GSK-3β was not that dramatic at 7 days after SE induction, the administration of lovastatin still decreased pGSK-3β/GSK-3β ratio (Figure 2B), to regulate the activity of CRMP-2. Moreover, according to our observation, in the absence of SE induction, lovastatin did not show any effect on the expression of GSK-3β and pGSK-3β, which further supported the idea that lovastatin regulated the expression of pGSK-3β and GSK-3β only after SE induction (Text S1 and Figure S1).

The expression of CRMP-2 was accordingly increased at both 3 and 7 day after SE induction (Figure 3), and lovastatin reduced the expression of CRMP-2, which is consistent with the moderation of GSK-3β/pGSK-3β ratio by lovastatin. The fact that lovastatin regulated GSK-3β and CRMP-2 is compatible to explain its anti-MFS effect. Therefore here we propose that the abnormal neuronal fiber growth in TLE animal model is possibly caused by SE induced GSK-3β phosphorylation. Although the relationship between the activation of GSK-3β/CRMP-2 and MFS is not well established, the fact that administration of lovastatin was able to reverse this effect and cease neuronal fiber growth still provided a potential target of statins and a new insight to further understand the underlying mechanisms of MFS and epileptogenesis, as the information provided by previous studies [9], [11]. Since lovastatin has not ever been reported as a specific agonist/antagonist as the other experimental agents, the triangular relationship between lovatstain, GSK-3β/CRMP-2 and MFS still needs further investigation. Furthermore, it is noticeable that a similar study has been reported that atorvastatin inhibits GSK-3β phosphorylation by cardiac hypertrophic stimuli [30], supporting our point of view that statins are able regulate GSK-3β after severe injury and plays a role in protecting cells from the further damages, in this case, MFS.

According to the results, at 7 days after SE induction, the expression level of GSK-3β was not significant with control group, indicating that this pattern was restricted within the first week after SE induction. In addition, a hypothesis that the subsequent pathophysiological alteration is likely initiated at the time of injury was proposed [31], [32]. Evidently, a previous study showed that the dramatic cytokinic alteration after SE induction take place acutely in the early phase and is thought dominant to determine the consequence of epiletogenesis [33]. Therefore, in our experimental design, we only examined the expression level of GSK-3β/CRMP-2 restricted to the first week after SE induction. The alteration for the later stage is waiting for further study.

### Neuro-inflammation and the Formation of MFS

Statins are effective neuroprotective agents in several brain pathological models due to their excellent anti-inflammatory activity [34]. After SE induction, several pro-inflammatory cytokines are activated or over-expressed rapidly, such as iNOS [35], COX-2 [36], tumor necrosis factors and interleukin families [33]. Drugs which inhibit these inflammatory molecules are reported to reduce seizure-induced damages [37], [38]. After SE induction, the inflammatory response surges rapidly and drops to basal level within 2 days [33]. Therefore, at the beginning of our experimental design, we hypothesized that if we administrated anti-inflammatory agents (lovastatin, L-NAME and dexamethasone) to block the primarily dramatic inflammatory response immediately after SE induction, which might be able to inhibit the downstream consequences to prevent or restrict the extent of MFS. In our experiment, anti-inflammatory drugs were injected subcutaneously 3 hours after the termination of SE (Figure 1A). However, the results did not support our hypothesis because L-NAME and dexamethasone neither show any significant effect on the expression and phosphorylation level of GSK-3β nor on MFS as well (Text S1, Figure S1 and 4).

A recent study reported that chronic administration of simvastatin alters the expression of IL-1, TNF but not IL-6 3 days after kainic acid-induced injury and further inhibits MFS [10]. However, there is no direct evidence to prove the causation of neuro-inflammation and MFS. In our study, we could not totally exclude role of neuro-inflammation in MFS by only single high dosage administration of anti-inflammatory agents since this regime might be not strong enough to terminate the robust activation of neuro-inflammation after SE induction. Besides comparing the response to anti-inflammatory agents (L-NAME and dexamethasone), an actual immune system modulation should be looked at to achieve a conclusions about the role of neuroinflammation.

### Lovastatin did not Mimic the Effect as NMDA Receptor Antagonist

Lovastatin is reported to reduce NMDA-mediated excitotoxicity on cultured cortical neurons [39]. NMDA receptor activation is a common pathway shared by different models of neurologic diseases. Most brain injuries are firstly ignited by the over-activation of NMDA receptor, which causes large amount of Ca^2+^ influx, injures the neuronal cells, initiates abnormal signals to further exaggerate cellular damages and recruits pathological alterations [40]. To block the activation of NMDA receptor immediately after brain injury is a well-recognized strategy to protect neuronal cells from further damage. In our study, both histological and electrophysiological techniques excluded the involvement of a direct NMDA receptor blocking effect of lovastatin since lovastatin did not showed any inhibitory effect on eEPSC_NMDA_ or GCD (Figure 6, 7).

### Pharmacological Significance and Clinical Implication

Our present data showed that lovastatin modulated the expression of GSK-3β and CRMP-2 and inhibited MFS. This seizure-oriented research added two major contributions to the current knowledge of neuropharmacology. First, although several pharmacological tools [2], [9], [11], and a few antiepileptic drugs [27] are reported to inhibit MFS, only a handful of them are clinically available. Our study extends the clinical application of lovastatin as an anti-MFS agent which is potentially helpful for treating epilepsy. Second, besides anti-inflammatory effect of statins, we revealed that GSK-3β and CRMP-2 pathway is also the target of statins, which provides a new insight to further understand the underlying mechanisms of MFS and epileptogenesis.

## Supporting Information

Figure S1 **Lovastatin, L-NAME, and dexamethasone did not significantly alter the expression and phosphorylation pattern of GSK-3β in the absence of pilocarpine-induced SE, determined by western blot.** The expression levels of (A) GSK-3β and (B) pGSK-3β were not changed by lovastatin, L-NAME, nor dexamethasone, at both day 3 and 7 after drug administration.
(TIF)

Text S1 **To examine whether lovastatin itself affects the total expression level of GSK-3β, we administrated lovatstain to the rats without SE induction and observed the expression level of GSK-3β.** Also we applied both L-NAME and dexamethasone to investigate the possible involvement of neuro-inflammatory pathway. In the lovastatin-treated group, the amount of GSK-3β did not show any difference between each group (control, 1.00±0.14; day 3, 0.99±0.11; day 7, 0.86±0.16, *P*>0.05, n = 3). In the L-NAME-treated group, the amount of GSK-3β did not show any difference between each group (control, 1.00±0.10; day 3, 1.01±0.02; day 7, 1.24±0.12, *P*>0.05, n = 3). In the dexamethasone-treated group, the amount of GSK-3β did not show any difference between each group (control, 1.00±0.11; day 3, 1.18±0.07; day 7, 0.90±0.03, *P*>0.05, n = 3). In the next we observed the expression level of pGSK-3β. In the lovastatin-treated group, the amount of pGSK-3β did not show any difference between each group (control, 1.00±0.17; day 3, 1.17±0.07; day 7, 0.90±0.09, *P*>0.05, n = 3). In the L-NAME-treated group, the amount of pGSK-3β did not show any difference between each group (control, 1.00±0.10; day 3, 0.99±0.03; day 7, 1.09±0.07, *P*>0.05, n = 3). In the dexamethasone-treated group, the amount of pGSK-3β did not show any difference between each group (control, 1.00±0.07; day 3, 1.08±0.01; day 7, 1.09±0.12, *P*>0.05, n = 3) (Student’s *t*-test)(Figure S1).
(DOCX)
