# Supplementary figures and images for: Lovastatin Modulates Glycogen Synthase Kinase-3β Pathway and Inhibits Mossy Fiber Sprouting after Pilocarpine-Induced Status Epilepticus
Source: PLoS One. 2012 Jun 26;7(6):e38789. doi: 10.1371/journal.pone.0038789 (PMC3383707; doi:10.1371/journal.pone.0038789)

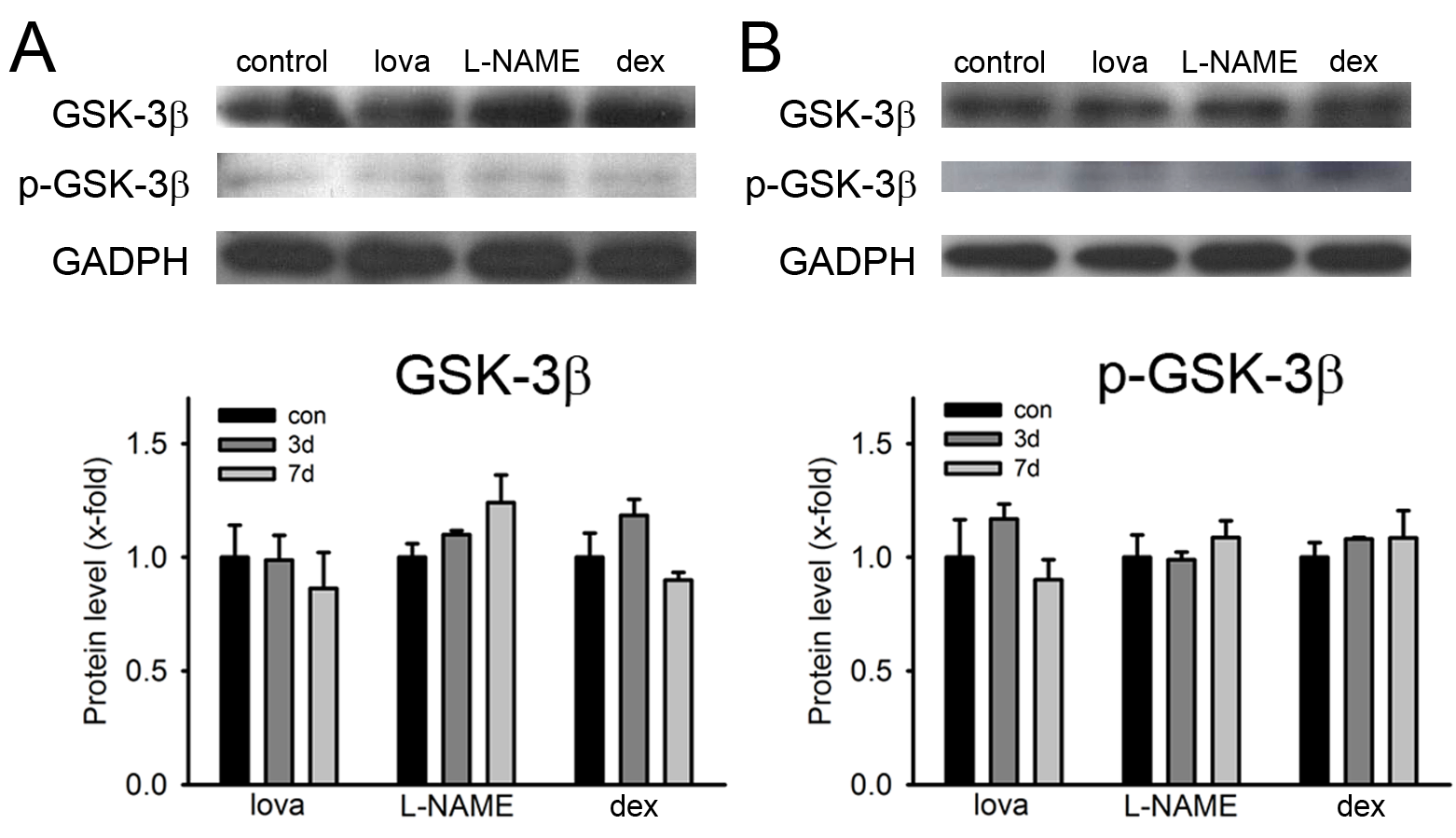

Supplement: Figure S1 — Lovastatin, L-NAME, and dexamethasone did not significantly alter the expression and phosphorylation pattern of GSK-3β in the absence of pilocarpine-induced SE, determined by western blot. The expression levels of (A) GSK-3β and (B) pGSK-3β were not changed by lovastatin, L-NAME, nor dexamethasone, at both day 3 and 7 after drug administration. (TIF) [file pone.0038789.s001.tif]
