# Supplementary material for: Lovastatin Modulates Glycogen Synthase Kinase-3β Pathway and Inhibits Mossy Fiber Sprouting after Pilocarpine-Induced Status Epilepticus
Source: PLoS One. 2012 Jun 26;7(6):e38789. doi: 10.1371/journal.pone.0038789 (PMC3383707; doi:10.1371/journal.pone.0038789)
Supplement: Text S1 — To examine whether lovastatin itself affects the total expression level of GSK-3β, we administrated lovatstain to the rats without SE induction and observed the expression level of GSK-3β. Also we applied both L-NAME and dexamethasone to investigate the possible involvement of neuro-inflammatory pathway. In the lovastatin-treated group, the amount of GSK-3β did not show any difference between each group (control, 1.00±0.14; day 3, 0.99±0.11; day 7, 0.86±0.16, P>0.05, n = 3). In the L-NAME-treated group, the amount of GSK-3β did not show any difference between each group (control, 1.00±0.10; day 3, 1.01±0.02; day 7, 1.24±0.12, P>0.05, n = 3). In the dexamethasone-treated group, the amount of GSK-3β did not show any difference between each group (control, 1.00±0.11; day 3, 1.18±0.07; day 7, 0.90±0.03, P>0.05, n = 3). In the next we observed the expression level of pGSK-3β. In the lovastatin-treated group, the amount of pGSK-3β did not show any difference between each group (control, 1.00±0.17; day 3, 1.17±0.07; day 7, 0.90±0.09, P>0.05, n = 3). In the L-NAME-treated group, the amount of pGSK-3β did not show any difference between each group (control, 1.00±0.10; day 3, 0.99±0.03; day 7, 1.09±0.07, P>0.05, n = 3). In the dexamethasone-treated group, the amount of pGSK-3β did not show any difference between each group (control, 1.00±0.07; day 3, 1.08±0.01; day 7, 1.09±0.12, P>0.05, n = 3) (Student’s t-test)(Figure S1). (DOCX) [file pone.0038789.s002.docx]

**Supporting information**

**Text S1.** To examine whether lovastatin itself affect**s** the total expression level of GSK-3β, we administrated lovatstain to the rats without SE induction and observed the expression level of GSK-3β. Also we applied both L-NAME and dexamethasone to investigate the possible involvement of neuro-inflammatory pathway. In the lovastatin-treated group, the amount of GSK-3β did not show any difference between each group (control, 1.00 ± 0.14; day 3, 0.99 ± 0.11; day 7, 0.86 ± 0.16, *P*>0.05, n=3). In the L-NAME-treated group, the amount of GSK-3β did not show any difference between each group (control, 1.00 ± 0.10; day 3, 1.01 ± 0.02; day 7, 1.24 ± 0.12, *P*>0.05, n=3). In the dexamethasone-treated group, the amount of GSK-3β did not show any difference between each group (control, 1.00 ± 0.11; day 3, 1.18 ± 0.07; day 7, 0.90 ± 0.03, *P*>0.05, n=3). In the next we observed the expression level of pGSK-3β. In the lovastatin-treated group, the amount of pGSK-3β did not show any difference between each group (control, 1.00 ± 0.17; day 3, 1.17 ± 0.07; day 7, 0.90 ± 0.09, *P*>0.05, n=3). In the L-NAME-treated group, the amount of pGSK-3β did not show any difference between each group (control, 1.00 ± 0.10; day 3, 0.99 ± 0.03; day 7, 1.09 ± 0.07, *P*>0.05, n=3). In the dexamethasone-treated group, the amount of pGSK-3β did not show any difference between each group (control, 1.00 ± 0.07; day 3, 1.08 ± 0.01; day 7, 1.09 ± 0.12, *P*>0.05, n=3) (Student’s *t*-test)(Figure S1).

**Figure S1.** Lovastatin, L-NAME, and dexamethasone did not significantly alter the expression and phosphorylation pattern of GSK-3β in the absence of pilocarpine-induced SE, determined by western blot. The expression levels of (A) GSK-3β and (B) pGSK-3β were not changed by lovastatin, L-NAME, nor dexamethasone, at both day 3 and 7 after drug administration.
